# Optimising web site designs for people with learning disabilities

**DOI:** 10.1111/1471-3802.12034

**Published:** 2013-10-22

**Authors:** Peter Williams, Christian Hennig

**Affiliations:** University College London

**Keywords:** Information technology, web design, learning disabilities, usability, eliciting preferences

## Abstract

Much relevant internet-mediated information is inaccessible to people with learning disabilities because of difficulties in navigating the web. This paper reports on the methods undertaken to determine how information can be optimally presented for this cohort. Qualitative work is outlined where attributes relating to site layout affecting usability were elicited. A study comparing web sites of different design layouts exhibiting these attributes is discussed, with the emphasis on methodology. Eight interfaces were compared using various combinations of menu position (vertical or horizontal), text size and the absence or presence of images to determine which attributes of a site have the greatest performance impact. Study participants were also asked for their preferences, via a ‘smiley-face’ rating scale and simple interviews. ‘Acquiescence bias’ was minimised by avoiding polar (‘yes/no’) interrogatives, achieved by asking participants to compare layouts (such as horizontal versus vertical menu), with reasons coaxed from those able to articulate them. Preferred designs were for large text and images. This was the reverse of those facilitating fastest retrieval times, a discrepancy due to preferences being judged on aesthetic considerations. Design recommendations that reconcile preference and performance findings are offered. These include using a horizontal menu, juxtaposing images and text, and reducing text from sentences to phrases, thus facilitating preferred large text without increasing task times.

## Introduction

Despite the near ubiquity of the Internet, there has been little research undertaken on its use by people with learning disabilities, and a paucity of empirical evidence regarding how to optimise web pages for information access and retrieval by this constituency. Even that which does exist is conflicting. Nielsen (1996) and Bohman (2010), for example, recommend avoiding the need to scroll. By contrast, other commentators (e.g., Keates, Adams and Bodine et al., 2007; Sevilla, Herrera and Martinez et al., 2007) urge the use of photos or other pictorial representation and video. Pages containing such content, however, tend to be longer, and therefore do require scrolling. This leads to the question of which attributes of web sites are the most important in designing for accessibility. The aim of the study was, therefore, to determine optimal web design for people with learning disabilities.

This question was addressed first within the context of a wider project: ‘Newham Easy Read’, a web site developed by researchers at the University of East London's Rix Centre, in conjunction with Newham Borough Council. It was further examined more experimentally using a series of web layouts created specifically to test various design elements. Web site content was based around ‘transition’, the phase in the lives of young people where they make the move from school to life in the community. The information content was developed with this cohort themselves, aided by the research team and their supporters (Minnion, Staples and Singh et al., 2008; Williams, 2008).

### The first ‘easy read’ site

**Figure 1:**
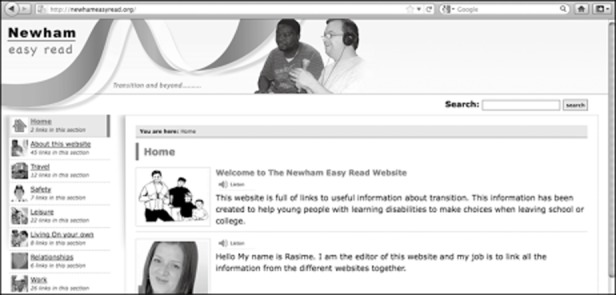
‘Newham Easy Read’ home page

The ‘home page’ of the original web site can be seen in Figure 1. Text content was kept to a minimum and is accompanied by an equivalent audio rendition. Images were also liberally used, as recommended in accessibility literature, such as that by the W3C (World Wide Web Consortium) (1999).

Usability studies on this site undertaken by the present writer (Williams, 2013; Williams and Hanson-Baldauf, 2010) involved the development of ‘one-action’ tasks suitable for people with low literacy skills. Observation of set tasks and free-browsing were undertaken, followed by simple feedback interviews. Of major interest was an examination of the barriers to effective information retrieval, with results feeding into the second part of the study. Factors that related specifically to the usability of the web interface included the use of images, text size and menu position. Each of these is described below.

*Images*: Many participants showed difficulties in interpreting the meaning of images depicting menu entries, and the more literate participants appeared to rely on the written caption to ascertain the topic depicted. Indeed, some of the images used could be interpreted in a number of ways even by ‘mainstream’ users. For example, that chosen to represent ‘support’ was a close-up of a handshake, which could have meant friendship, some kind of agreement or even a ‘goodbye’. Also, image use necessarily results in longer pages, often extending below screen level and so requiring scrolling. This was a big issue for some participants, with the least able appearing not to realise that ‘invisible’ content existed, some not being able to scroll effectively, and others not inclined to do so.
*Text size*: Although study participants tended to prefer larger font, and some guidelines emphasise the importance of making it possible to increase font size (e.g., British Standards Institution, 2006), the space taken up by even a small body of large text pushes the page length down below the visible screen. However, in print form, larger text sizes have been shown to be more readable than smaller sizes (Mills and Weldon, 1987; Rudnicky and Kolers, 1984; Tinker, 1963), although the differences are often not significant until the size differential becomes quite large. However, Michael Bernard and colleagues (Bernard, Lida and Riley et al. 2002) noted that no significant research had been undertaken on text size in an online or technology environment. This still appears to be the case in 2013.
*Menu position*: Clearly, access to content needs to be as simple and accessible as possible (and not just for people with learning disabilities!), and so the presentation of menu entries is of great importance. The preliminary qualitative work suggested that, for these users, the horizontal option might be the optimal choice. Two possible reasons are that the entire menu is visible on screen (discounting horizontal scrolling, heavily frowned upon by accessibility experts, such as Burzagli, Emiliani and Gabbanini, 2009; Colorado and Eberle, 2009), and the direction in which the information presented is compatible with the eye movement of someone reading. In other words, it may be easier for someone with learning disabilities to read menus from left to right rather than vertically. Interestingly, prior research (e.g., Bernard and Hamblin, 2003; Tullis, 2005) appears to suggest, by contrast, that a vertical menu aids information retrieval more effectively than, for example, ‘drop-downs’ or ‘fly-outs’. These studies, however, were with mainstream participants. Whether this is the case for people with low levels of literacy has yet to be examined. Also, the number of menu entries used in ‘Newham Easy Read’ was problematic, not only because negotiating the entries required scrolling, but also because they were very small and simply too numerous to take in.

### The experimental ‘Pete's Easy Read’ site

#### The structure and content of the site

**Figure 2:**
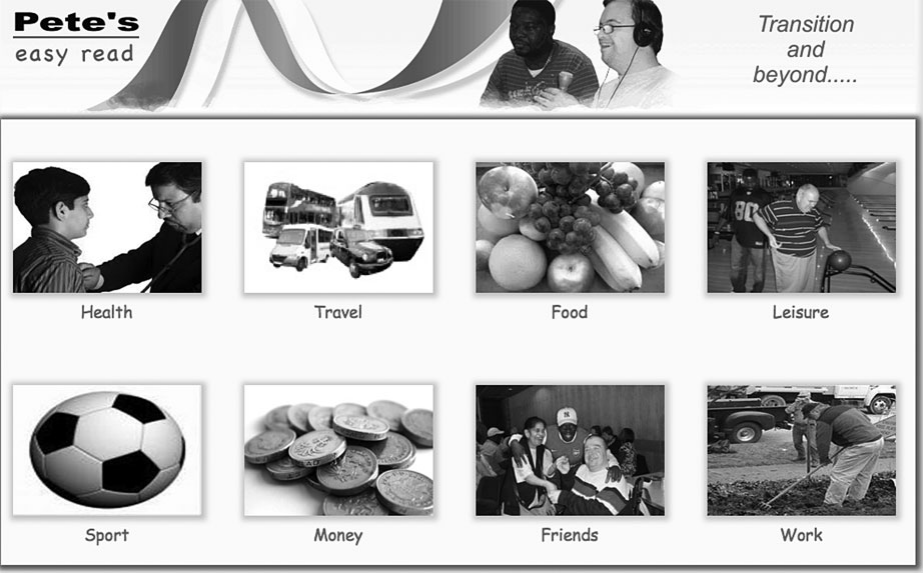
‘Pete's Easy Read’ home page

This web site included various design modifications suggested by results from the original project, and facilitated comparison of the attributes mentioned above. Figure 2 shows the ‘home’ page. The grid menu design was present only for this ‘portal’ page. Vertical and horizontal arrangements were tested for the information pages themselves.

**Figure 3:**
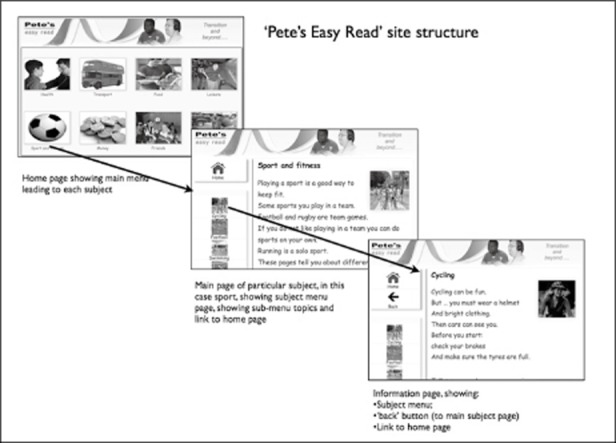
‘Pete's Easy Read’ site structure

As with ‘Newham Easy Read’, the site contained three levels (see Figure 3):

- The home page, as shown in Figure 2, giving access to each of eight main topic pages;
- A subject menu page itemising information pages on each topic, and unlike the original site giving a 50-word topic introduction; and
- Information pages on each of the subtopics.

Eight interface designs accrue from the attributes under scrutiny, as shown in Table 1.

**Table 1:** Interface designs used

Interface number	Contents position	Images (yes/no)	Text size
Interface 1	Horizontal	No	Small
Interface 2	Horizontal	No	Large
Interface 3	Horizontal	Yes	Small
Interface 4	Horizontal	Yes	Large
Interface 5	Vertical	No	Small
Interface 6	Vertical	No	Large
Interface 7	Vertical	Yes	Small
Interface 8	Vertical	Yes	Large

**Figure 4:**
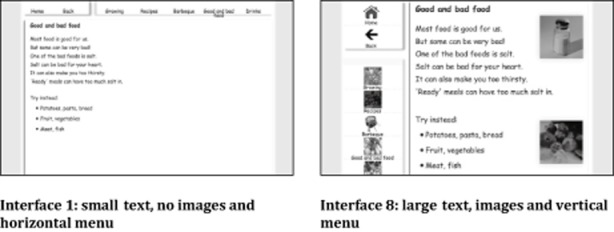
Information page on ‘good and bad food’, as presented by two different interfaces

By testing information retrieval performance on each of these interfaces, it was possible to address the question of which attributes of web sites are the most important in designing for accessibility, taking into account the effect that each variable has on the overall site appearance and functionality. Two examples of the different layouts can be seen in Figure 4.

Far fewer entries were included here (five), following earlier findings regarding scrolling and problems negotiating a large number of items. Navigation consisted of simply ‘home’ and ‘back’ buttons.

## Methodology

### Participants

The study was undertaken at various locations, including the special needs unit of a college of further education, an adult education class, a self-advocacy group and adult day centres. Participants had levels ‘entry two’, entry three’ or ‘level one’ of literacy, as used in UK Further Education colleges (Moser, 1999). As such, they could be expected to do the following:

- Read and understand simple text (those at entry three, up to six sentences in one paragraph);
- Get the main idea from a simple graphical or tabular source (e.g., safety signs); and
- Understand and use a simple list.

In total, 104 people participated in the study, ranging from 17 to 63 years of age, 94 of whom undertook sufficient number of tasks to be included in the analysis. It is important to note also that participants had no physical disabilities. Thus, they were all able to manipulate a touch pad or mouse normally, and were not visually impaired.

### Method

Unlike with the original exploratory usability work, this phase of the research used quantitative methods, involving a statistically significant number of participants and recording task time. Regarding this measure, it could be argued that the time taken to access information does not matter. However, there are a number of reasons for adopting this measure:

- *The nature of the task:* The usability element of the study looked at retrieving information rather than creating or communicating it. As such, speed of access may be more important.
- *Short attention span:* People with learning disabilities are known to have short attention spans (Learning Disabilities Association of America, 2010), and time may be important in terms of willingness to engage with an information resource.
- *Precedent:* Time on task is a standard measure in usability testing (see, e.g., Choi and Bakken, 2010), including those addressing issues related to people with learning disabilities (see, e.g., Karreman, van der Geest and Buursink, 2007).

Participants undertook tasks on the eight interface configurations of the new web site, one task per interface, presented at random. Tasks required participants to do the following:

- Recognise and activate a hyperlink (from a text label, with or without an accompanying image);
- Recognise the need to scroll a page as appropriate and know how to do so;
- Read simple text; and
- Identify a string of text containing the answer to a simple question.

**Figure 5:**
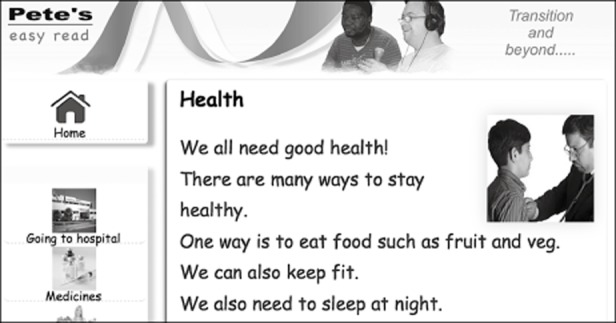
Subject ‘home’ page for health

**Figure 6:**
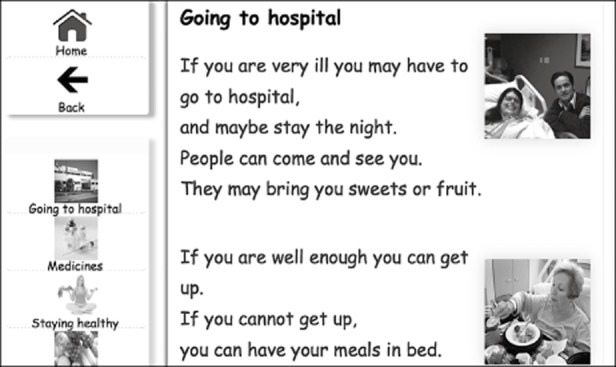
Information page: ‘Going to hospital’

Four steps were involved in each task, with the goal being to extract two pieces of information from each of the main subject sections, as follows:

*Step one*: Identifying the subject from a main grid menu on the ‘home’ page.
*Step two*: Answering a question from the introductory paragraph of a particular subject (Figure 5 shows the ‘subject home page’ for health in which this page sits).
*Step three*: Identifying the within-subject topic from the subject menu (Figure 5 again), requiring recognition and activation of a hyperlink, on occasion below screen level, and thus requiring scrolling.
*Step four*: Answering a question from the text of the topic accessed. Figure 6 shows the information page for ‘going to hospital’, required an answer to one question.

It is worth noting here that these subtasks illustrate the manner in which electronic ‘reading’ entails a more complex activity – even for the relatively simple exercise undertaken here – than negotiating the print medium. Don Leu of the University of Connecticut (e.g., Leu, Kinzer and Coiro et al., 2004; Leu and Zawilinski, 2007; Leu, Zawilinski and Castek et al., 2007) is a leading figure in ‘new literacies’ occasioned by electronically presented information, and points out that although, for example, pictures, charts and maps are present in the print environment, the nature of Internet multimedia poses ‘unique problems … In an electronic environment, decoding for comprehension includes decoding the strategic use of color (sic); various clues that indicate hyperlink(s); … icons and animations … that are not static’ (Leu et al., 2004, p. 1582). In the current case, the major attribute to be negotiated not present in print form are the hyperlinks and their navigation.

**Figure 7:**
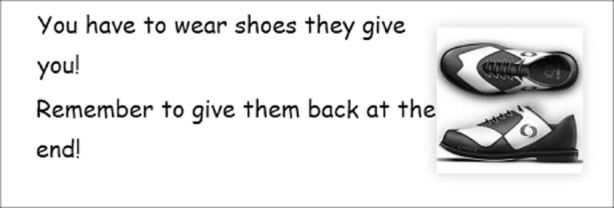
Information page showing the juxtaposition of text and image

Issues arising in the set tasks were as follows:

*Question formulation*: Questions were worded in such a way as to not require participants to infer answers or break text down to elicit information. This is because the study sought only to examine the effects of web design on information retrieval, and not comprehension. Thus, questions were used whose answers contained the same words as those in the question. For example, ‘Where can you have your meals?’, where the text includes the phrase ‘you can have your meals in bed’.
*Juxtaposition of image and text*: One of the research questions was that of whether images aid information retrieval. Figure 7 shows the juxtaposition of text and image related to the question ‘What do you have to wear to go bowling?’ The images were chosen both to illustrate and make the text easier to follow, and to test whether the images helped participants arrive at the answers to questions.
*Positioning of sought content*: One final feature of the tasks is that of text and image positioning. Clearly, it might take a longer time to find information if it is situated at the bottom of the screen, and even longer where that necessitates scrolling. To concentrate the study more on the effects of the conditions under consideration rather than on that of scrolling *per se*, where the required menu entry item (step three) appeared at the bottom of the (vertical) list of entries, the information sought in step four would be near the top.

### Data gathering and analysis

Two types of analysis were used to compare interface designs:

- A statistical analysis based on measures of task time; and
- A qualitative analysis comprising researcher observations and interactions with participants, and post hoc informal interviews with participants and carer/tutors, etc.

With regard to the statistical analyses, it is important to note that in the dataset, several observations come from the same test person. Strictly speaking, these observations should not be treated as statistically independent because the individual characteristic of the test persons will affect all their observations. This was addressed statistically by using a number of ‘mixed-effects models’ (Pinheiro and Bates, 2000; R Core Team, 2012). Such models take into account the within-test persons dependence (as outlined above) of observations and allow to test the factors of interest while taking into account all other factors present.

Post-test interviews were undertaken to seek preference data regarding the interfaces presented. An issue here was the documented propensity of an interviewee to agree with someone in apparent authority. Meisenberg and Williams (2008) found that ‘less educated and intelligent’ people tend to be susceptible to this ‘acquiescence bias’. Space does not permit a full consideration here, but in short, polar (‘yes/no’) interrogatives (‘Do you like the layout?’) were eschewed, with one way of doing so being to ask for comparisons (such as horizontal versus vertical menu), with reasons coaxed from those able to articulate them.

## Results

### Testing for validity

It was important to test whether there was a significant difference in task difficulty and in task time with regard to task order. Care was taken to ensure each task was as equal as possible, by site pages each being written at the same reading level, avoiding inference questions and obtaining feedback from professionals regarding language level and equality of questions. Analyses showed that neither task topic (*P* = 0.45) nor task order (*P* = 0.10) was significant, suggesting that the results could be attributed to the factors being investigated and not to other factors in play.

### Individual site attributes and performance

Each attribute (or variable) of the web site was looked at in isolation. In each case, there were two cases for each attribute. These were the following:

- Presence or absence of images;
- Horizontal or vertical menu; and
- Large or small text size.

Turning first to the use of images, it is important to state that the selection used was undertaken after working with people with learning disabilities themselves, and with the expert help of professionals in the field. Thus, they were as representative as possible. However, comparing the presence or absence of images suggested no correlation between task times for pages displaying images or not. Thus, images appeared to offer little or no help in accessing information – at least not in terms of speed of access. If this is considered a surprising result, a study by Poncelas and Murphy (2007) came to a similar conclusion – in their case that the addition of symbols to simple texts does not necessarily improve people's understanding of it.

Observational findings suggested one major reason for this. Participants appeared to be focused almost exclusively on text-based content in order to undertake the tasks, ignoring what they may have considered to be extraneous detail because they had to concentrate so hard on the text. Summers and Summers (2005) also noted this. In hindsight, an argument could be made for dispensing with text almost altogether, so that, in the page on bowling, ‘You have to wear shoes they give you!’ might become ‘wear these!’ next to an appropriate image, as discussed in more detail in the Recommendations section, below.

With regard to menu position, vertical menus significantly increased task time (*P* = 0.001). The difference only corresponds to a mean increase of 5.56 seconds per task, but considering that there were only five menu entries on each page, one can assume that the increase in time would be far greater with a ‘conventional’ site. Indeed, even sites written for this constituency are prone to having a large number of menu entries. Dobson's Choice, 1 for example, had 15 on 22.04.13; and ‘Movingonup 2 ’ has eight always-visible menu entries, plus the same number on each of the main subject pages.

The finding contrasts with other (albeit not equivalent) studies, such as that described earlier by Ojanpää and colleagues (Ojanpää, Näsänen and Kojo, 2002), although this is in line with that of Laarni, Simola and Kojo et al. (2004), who studied reading rather than word search. To explain the results, it is worth noting that some participants, despite being asked to look at the menu (and shown its location) tended, instead, to read through the text. Even gentle probing by the researcher failed in these cases to determine whether this was because of a poor understanding of the task or a belief that the link was embedded in the text. In fact, there were cases where it was not obvious whether the menu or the text was being consulted. Importantly, this behaviour appeared to affect performance on the vertical menu pages more than on the horizontal – seemingly because when one reads, one does so from the top of the page, which is where the horizontal menu was placed.

Following on from this, the propensity to absorb information methodically from top left to bottom right suggests that where there is distracting material present, the vertical menu entries are accessed more slowly than the horizontal ones. It appears that the lower entries of the vertical menu were only accessed after horizontal sweeps of the entire page.

The final layout/design variable considered was text size. There was a difference in task time between the two text sizes presented (*P* = 0.006). This corresponded to an increase of 5.06 seconds in time for an average user – with the larger text size taking longer. Not surprisingly, large text size pushed the content to below screen level (as did images), and so in a minority of cases the task answers were not visible on the page. Not surprisingly, visibility as a variable proved significant (*P* = 0.02), controlling for which (e.g., excluding answers that were not visible) resulted in text size no longer being significant (*P* = 0.21). This means that the effect of text size can be explained by its effect on visibility. Nevertheless, one cannot discount this – because the very fact that text size does have this effect means that information will take longer to find from pages where the text is particularly large, and longer still where more text exists.

This result contradicts traditional guidelines (e.g., Bohman, 2004; Hassell, 2005) that state that a larger text size should be used, although, as mentioned earlier, the present participants were not visually impaired. Observations suggested that the longer time taken to read large text was a result of two factors:

- The increased number of lines generated by large text; and
- The resulting increased length of the page.

The increased number of lines was particularly manifest in the vertical menu condition, as the width of the area of the page occupied by the body text was reduced. The ‘with-images’ condition further reduced line length, unlike in the small-text condition, as the short sentences generally meant that one sentence fitted on one line even where the space was reduced. In negotiating large text, readers had to focus more on moving from one line to the next, and those who read aloud tended to pause between lines. This may have made it more difficult to read efficiently and with good understanding.

This study limited its scope to ‘computers’, as in desktops and laptops. Clearly, a new set of issues arise when talking about mobile devices, such as smartphones, etc. With the screen so small, the issue of text size and scrolling becomes more important. Also, the position of the screen relative to the user may be different, and the data entry system touch screen rather than mouse and (physical) keyboard. There is also the increasing availability of mobile text to speech, raising the issue of the effectiveness of audio as an information medium, an issue discussed more fully in an earlier paper by one of the present writers (Williams, 2013). As Clayton Lewis and colleagues (Lewis, Sullivan and Hoehl, 2009, p. 387) point out, ‘[a]s smart phones become more powerful, they offer the possibility to translate complex information into simpler, more comprehensible forms that are appropriate to an individual's abilities’. Indeed, Joseph Mintz and colleagues (Mintz, Branch, March et al. 2012) have recently developed mobile phone software to help develop social and life skills in children with autistic spectrum disorders.

### Combined site attributes and performance

**Figure 8:**
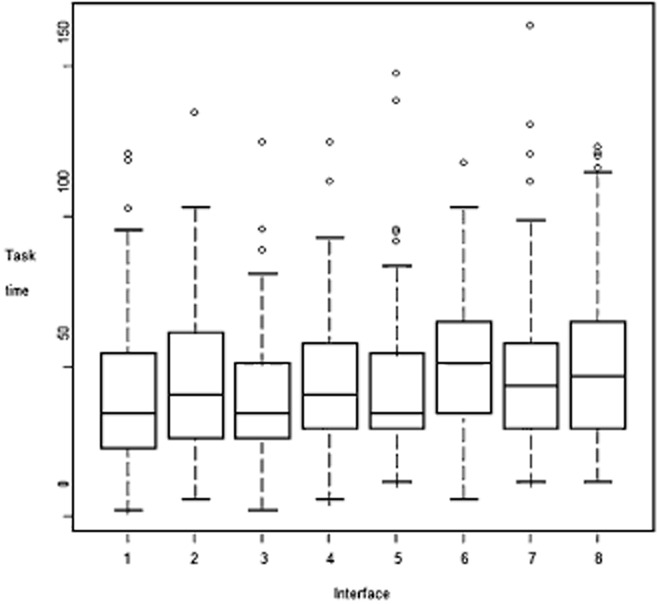
Box plots of the task times of the eight interfaces

Taking first the ‘performances’ of each interface, Interface One (horizontal menu, no images and small- text) is the fastest, and Interfaces Six (vertical, no images and large text) and Eight (vertical, with images and large text) are the slowest. Box plots of the data are shown in Figure 8. In the box, there are 50% of the data, 25% each in the part above and below the horizontal line, which represents the median value. The end of the dashed ranges is the smallest/largest observation in the data that are not classified as outlier, the latter are shown as individual observations above or below the dashed range.

Interaction between the basic variables (e.g., text size, menu position and presence of images) was also measured. This was tested by a conditional *F*-test (Pinheiro and Bates, 2000). There was no significant interaction between the variables (*P* = 0.832), suggesting, perhaps surprisingly, that changing one (e.g., text size) does not affect the contribution of any other variable to task time.

## Discussion

Overall, the results accrued do not necessarily support standard guidelines. This is particularly true with regard to the finding that small text was more effective in information retrieval and the ineffective use of images. The results can be explained by reference to observational findings. The most important of these is that many of the participants elected to read rather than merely skim the text. In many cases, this practice was clear. A minority read aloud, some followed the text with the curser, and a smaller group read with their finger on screen guiding them. This group, clearly, read linearly and made no effort to scan the text either to search the text or to look for non-verbal clues (in this case the images) to help. Others also appeared to be reading word for word, simply from their eye and head movements, although in these cases the indications were less certain, as such movements, although suggestive, did not provide proof of reading. Some prior literature has also noted this tendency (Summers and Summers, 2005; Theofanos, Mulligan and Redish, 1963), which would explain why participant performance did not improve with practice. Even when undertaking the last task, they would begin at the top and work slowly downward linearly.

It can be said from this that the participants acquired information by ‘serial access’. Just as in a computer, serial access to data is that where the data are read from the storage medium in the order in which they were recorded until the required item is reached (Daintith and Wright, 2010). Although not used in the field of education, one might use the example of multiplication tables, where the product of six sevens might only be accessible by going through the sequence ‘two sevens are 14; three sevens are 21 …’, etc. until the required equation is reached. The lack of significant difference in task time with regard to task order also suggests such ‘serial access’, as participants did not appear to learn to skim-read or use images to find information as their experience of undertaking each task increased.

Although the phenomenon of serial access may sound like a synonym for ‘linear access’, a phrase that is common in web design and usability (see, e.g., Horton, 2005), it is important to note that the terms do not mean the same thing. Linear access (i.e., accessing information from left to right, and top to bottom) includes skimming, the speed reading of content facilitated by omitting trivial words while searching for a key fact or phrase or other item of content. This study presents evidence that people with learning disabilities not only access content linearly, but that they also indiscriminately imbibe all the information as they proceed. This is the defining characteristic of ‘serial access’.

This finding is in contrast to previously observed web behaviour, both by the present writer (Williams and Hanson-Baldauf, 2010) and others (Wilkinson and Payne, 2006), and also to behaviour observed during the qualitative phase of this work, in sessions where participants were given free rein to look at web resources. Behaviour under these circumstances shows high levels of skimming, scanning and browsing. For mainstream Internet users, the practice appears to be prevalent over a wide span of web activity, including that of specific information seeking (Nicholas, Rowlands and Williams et al., 2011) For the present participants, however, although indulging in apparent skimming and much rapid jumping from page to page during ‘free browsing’, this ceased when the activity changed to negotiating specific tasks. Clearly, solving information tasks represented a qualitatively different activity than that of free browsing, and one that was far more intellectually challenging.

In short, serial access helps explain results found regarding each of the attributes. Thus, images did not help find information as they were ignored until reached serially; and small text proved more effective than large (the participants were not visually impaired), as it required fewer lines, and therefore fewer eye movements. The more effective menu position was horizontal, partly because page contents laterally juxtaposed with vertical menus proved distracting, but also as it may have been difficult when reading the text body serially to ignore menu entries in the side column.

### Preference findings

Before discussing the findings, a note of explanation is required regarding the figures given. A total of 43 people gave their preferences. Time constraints, fatigue, and in a minority of cases, a straight declining of the invitation to give views prevented all of the participants from undertaking this element of the study. The horizontal menu was preferred by a small margin over the vertical one (18–11, with 14 offering no preference), with comments indicating it was ‘a bit easier’ and ‘It goes across’ (to which comment the researcher asked whether this meant it was easier, 3 obtaining an affirmative reply). Similarly, the large-text condition (preferred by 29 people to 6, with 8 ‘no- preference’) was described as being ‘easier to read’, ‘better’ and ‘nice’. The small-text condition was simply ‘too small’, although one person who rated it as ‘like a lot’ said it was actually easier to read, as it was ‘all in the same place’. Interestingly, being able to see more text in one saccade or eye movement and, complementary to that, not having the text extend too low on the page were reasons given by professionals, when they were shown results suggesting that the small-text condition interfaces performed better in terms of quicker access to information.

The images attribute stimulated more comments than the other two. Only one participant of 43 preferred pages without images. Most participants said images made the page look ‘nice’, ‘gives it colour’, ‘helps you to understand’ and ‘makes the writing easier’. The participant who did not like the images said that he did not like the particular picture shown. He was not asked whether he would like the pictures illustrating another page, for fear of provoking a positive response merely to please the researcher (i.e., the ‘acquiescence bias’ mentioned earlier). Two of the (five) participants who rated both image conditions the same made remarks to the effect that, as one of them declared, ‘it doesn't matter whether there are pictures – I can read’.

In comparing these results with those related to performance, there is only partial agreement between attribute preferences and attribute performances in terms of task time. The clearest agreement was in relation to menu position, where the preference for a horizontal positioning reflected findings that information was accessed quicker from sites with that arrangement. Results regarding text size were opposed, with participants stating a preference for large text even though information from the small-sized text was accessed quicker. The positive comments and ratings for the use of images is not surprising, despite that fact that they appeared to play little or no part in information retrieval.

## Recommendations

When undertaking a study that includes vulnerable people as participants, it is especially incumbent upon the researcher(s) to formulate recommendations that may be of real practical benefit to and for project participants, and indeed the population from which they are drawn. Any such recommendations from the present study need to address two major considerations. These are reconciling preferences versus performances, and obviating problems inherent in ‘serial access’ behaviour. Of course, the caveat needs to be made that, as mentioned above, people with learning disabilities, like everyone else, have varied and individual needs and abilities. These recommendations, therefore, should be seen as rough guides only, to be tailored and adapted for any specific known user group. Similarly, they are not aimed at one particular group, such as web developers, teachers or information providers. The interplay between text and images, for example, might be decided by a teacher and put into action by a web developer; the text density and level might be decided by an information provider.

The considerations of performances and preferences can be addressed together by looking at the interplay between text and images. The use of pictorial representations, unsurprisingly, was very popular despite their ineffectiveness in terms of information retrieval times, observationally shown to be due to pages not being examined globally. Even with only around 50 words to negotiate, participants had to concentrate so much on the text, and thus consuming it ‘serially’, that they did not engage with the other elements of the page. This clearly suggests that cutting text even from this modest word count may be advisable. An attempt to do this can be seen in Table 2, which shows two versions of a page on the leisure activity of bowling. The original text is on the left, with an edited version on the right.

**Table 2:** Full (left) and edited (right) versions of the page on bowling

Bowling is really fun!	Bowling is fun!
You need at least two people to play.	Two or more people to play
Before you go bowling you need to know	You need to know. …
• where the bowling rink is	• Where it is
• how to get there	• How to get there
• and how much it costs.	• Cost
You have to wear shoes they give you!	Wear shoes they give you
Remember to give them back at the end!	Give them back after!

The shorter version has been cut such that it still includes virtually all the information content of the original. The text could be reduced even further, in fact, although some of the information would be lost. However, using supporting images and juxtaposing these with the text may both aid understanding and speed of retrieval, and also address the strong participant preferences for images. Reducing the text content would also resolve the discrepancy between preferred text size (large) and most efficient in terms of information retrieval (small). With fewer than around 20 words per page, the length is unlikely to creep below screen level, and the slightly longer time it may take to read large text would be minimised.

Both performance and preferences matched with regard to menu layout. Notwithstanding the fact that only two conditions were explored, a horizontal menu appears to be easier to use, certainly in pages that also contain a body of text – and preferable with regard to serial access and expressed preferences. However, the menu list should be clearly distinguishable from the body text, by a border. Of course, one potential problem with a horizontal arrangement is that, even if the menu spilled onto two rows, the number of possible entries would be limited. However, research undertaken with the ‘Newham Easy Read’ site showed that many menu entries could be confusing. If possible, therefore, small discrete web sites may be the answer where necessary.

In sum, possible design recommendations, considering both performance and preferences, could consider the following:

- The organisation of text-based information such that the most important content is at the beginning. This is a common suggestion (see, e.g., Loranger and Nielsen, 2006) and is practised religiously in journalism, where it is known as the ‘inverted pyramid’ (e.g., Pottker, 2003), and particularly relevant in the current context, considering the restrictions of ‘serial access’ outlined earlier.
- Ensuring the juxtaposition of text and images (and, of course, the relevancy of the image to the text).
- Minimising word count and text density to reduce or maintain short page length.
- Using a fairly large text size, assuming a minimum amount of content (browsers can be configured to display the size of one's choice, but only five examples were noted in this research of a browser or desktop adjusted for individual use).
- Designing a menu layout where all of the entries are clearly visible on the page and, considering both performance and preference findings, horizontally arranged.
- Accompanying images will not automatically aid comprehension. The research showed how difficult it was to match, in particular, abstract concepts.
- With regard to the ambiguity of images, potential users could be consulted so as to arrive at some kind of consensus about the most appropriate representations. Of course, continued exposure to and consequent familiarity with a resource would in time help users learn what represents ‘health’, ‘support’, etc.

Of course, there are other considerations too, such as those of page width, font type, colour combinations, etc.,– moving from web page layout to web site layout, and the attendant issues around structure and navigation. These were, however, beyond the scope of this study.

## Conclusion

This study sought to compare different interface designs produced following qualitative work outlined briefly at the start of this paper, and in more detail in Williams (2013) and Williams and Hanson-Baldauf (2010). The research elicited one very important aspect of information retrieval behaviour that impacted in unexpected ways on performances with regard to interface design. This was the practice of ‘serial access’ to content when undertaking the set tasks, which contrasted strongly with the ‘random access’ behaviour of rapid consumption and skimming of content and general superficial behaviour when not seeking specific information. It also showed a marked contrast between page designs that facilitated information retrieval and those that were preferred by the project participants. Recommendations that accounted for serial access and reconciled performance and preference designs were offered.

It is appropriate to conclude this paper with a final word about the aspirations of self-advocacy, inclusion and equality. As mentioned, the provision of accessible, relevant and timely information is one way that can facilitate these aspirations [DH/CNO (DH Partnerships for Children, Families and Maternity/CNO Directorate), 2008]. Many of the findings outlined in this paper contradict current guidelines, so hopefully they will inform a debate currently lacking empirical evidence around how best that information may be presented. If it has helped the individuals who were kind enough (and brave enough!) to participate in this research, and the small minority who were too shy or too engaged in other more compelling activities, to better access and engage with information, then it will have been worthwhile.

## Footnotes

1 http://www.dobsonschoice.co.uk/

2 http://www.movingonup.info/

3 And in doing so being unable to prevent himself from asking the ‘polar interrogative’ he tried so hard to avoid!

## Authors' note

Pete Williams developed the research, devised and carried out the fieldwork. Christian Hennig undertook the statistical analysis.
